# Efficacy of Acupuncture in Itch: A Systematic Review and Meta-Analysis of Clinical Randomized Controlled Trials

**DOI:** 10.1155/2015/208690

**Published:** 2015-04-30

**Authors:** Chi Yu, Pei Zhang, Zheng-Tao Lv, Jing-Jing Li, Hong-Ping Li, Cai-Hua Wu, Fang Gao, Xiao-Cui Yuan, Jing Zhang, Wei He, Xiang-Hong Jing, Man Li

**Affiliations:** ^1^Department of Neurobiology, Tongji Medical School, Huazhong University of Science and Technology, Wuhan, Hubei 430030, China; ^2^Department of Orthopedics, Tongji Hospital, Tongji Medical College, Huazhong University of Science and Technology, Wuhan, Hubei 430030, China; ^3^Institute of Acupuncture and Moxibustion, China Academy of Chinese Medical Sciences, Beijing 100700, China

## Abstract

*Background*. Itch (pruritus) is a sensitive state that provokes the desire to scratch. It is not only a common symptom of skin diseases but it also occurs in some systemic diseases. Clinical studies on the efficacy of the acupuncture therapy in alleviating itch are increasing, while systematic reviews assessing the efficacy of acupuncture therapy are still lacking. *Objective*. This systematic review aims to assess the effectiveness of acupuncture therapy for itch. *Materials and Methods*. A comprehensive literature search of eight databases was performed up to June 2014, and randomized controlled trials which compared acupuncture therapy and placebo acupuncture or no treatment group were identified. Accordingly, a meta-analysis was conducted. *Results*. This review included three articles of randomized controlled trials (RCTs) from a total of 2530 articles. The results of Meta-analysis showed that acupuncture therapy was effective to alleviate itch compared with placebo acupuncture and no treatment group. *Conclusion*. Based on the findings of this systematic review, we cautiously suggest that acupuncture therapy could improve the clinical efficacy of itch. However, this conclusion needs more studies on various ethnic samples to confirm our final conclusion.

## 1. Introduction

Itch (pruritus) is an unpleasant cutaneous sensation which provokes the desire to scratch. It can be divided into acute and chronic itch [1]. Pruritus induced by chronic itch is not only a common symptom of skin diseases but also occurs in some systemic diseases, which may last for more than six weeks and seriously reduce the quality of patients' lives [2]. In 2007, the International Forum for the Study of Itch (IFSI) proposed a clinically oriented classification scheme consisting of 6 categories [3]: (1) dermatological (atopic dermatitis, psoriasis, etc.), (2) systemic (kidney dialysis, liver cholestasis, etc.), (3) neurological (postherpetic neuralgia, etc.), (4) psychogenic (e.g., delusional parasitosis), (5) mixed (overlapping and coexistence of several diseases), and (6) others (undetermined origin) [4].

Itch is widespread and is usually treated by pharmacological therapies. Western medicines such as antihistamines are the reference treatment, but they are generally ineffective in treatment [5]. Antihistamines may relieve histamine-evoked acute itch but they do not lessen chronic itch caused by skin, liver, or kidney diseases [5]. In addition, creams containing local anesthetics [6], capsaicin, doxepin [7], strontium nitrate [8], or nedocromil sodium [9] can be helpful to relieve itch. However, it is impossible to apply them to a large area of skin. The long-term external application of glucocorticoids will bring several side effects such as dry skin and atrophic skin [10].

Complementary and alternative medicine (CAM) is widely advocated to face the increasing demand for nonpharmacological approaches. As a mainstream CAM therapy, acupuncture treatment based on Traditional Chinese Medicine theory has been commonly used to treat itch for over 2,500 years [11]. Recently, clinical studies on the acupuncture therapy to alleviate pruritus are increasing. Several clinical trials have demonstrated the therapeutic effects of acupuncture therapy on pruritus, such as acupuncture, moxibustion, or pressing acupoint [12–14]. Moreover, placebo controlled studies have shown that acupuncture can reduce histamine induced acute itch in healthy human adults [15, 16] and chronic itch patients such as uremic pruritus [17, 18], type I hypersensitivity [19], atopic dermatitis [20], and neurogenic pruritus [21].

Unfortunately, systematic reviews assessing the efficacy of acupuncture therapy in the treatment of itch are still lacking. Thus, the aim of this systematic review is to perform a systematic literature search of all published RCTs and to compare the efficacy of acupuncture with placebo acupuncture and no treatment group.

## 2. Materials and Methods

### 2.1. Search Strategy

We searched the following western databases until June 2014 to identify trials: PubMed, Web of Science, Embase, Cochrane Library, and Medline. In addition, we searched the Chinese databases, such as the China Knowledge Resource Integrated Database, Wan Fang Database VIP Database, and Chinese Biomedical Literature Database. All of these databases were searched from their available dates of inception to the latest issue up to June 2014 [22].

Different search strategies were combined as follows. After searching the MeSH Database, we chose free text terms, such as “itch,” “itching,” or “pruritus,” which are English synonyms of itch. For western database, we used search strategy as follows: (“itching” OR “pruritus” OR “pruritis”) AND (“acupuncture” OR “acupoint” OR “moxibustion” OR “acupressure”) AND “randomized controlled trial.” For Chinese database, we search (“Zhen” OR “Jiu” OR “Xue Wei” OR “Zhi Ya”) AND “Yang.” To collect enough tests, related publications list of references are determined by searching for additional research. We also searched trials registers, hand-searched conference proceedings, checked the reference lists of all included and excluded articles, and contacted Chinese medicine experts for unpublished studies.

### 2.2. Selection Criteria

Inclusion criteria are as follows. (1) All the articles should be restricted to skin itch and must include the analysis methods and the degree of itch and the area of skin lesion. (2) The articles should be related to clinical trials. (3) All the articles should include randomized control trails (regardless of blinding, publication status, or language). Exclusion criteria are as follows: (1) Animal experiments. (2) Articles without randomized control trails such as retrospective studies, reviews, and case reports. (3) Other organs' itch, such as the nose itch, throat itch, and eye itch. (4) Some peculiar therapy like bee venom acupuncture. (5) Without related data to evaluate itch.

### 2.3. Outcome Assessment

For all the three included studies, we screen multiple indicators of itch such as mean itch intensity, Eppendorf Itch Questionnaire (EIQ) ratings, efficacy, wheal and flare size, and skin perfusion [17, 19, 23]. Eventually, only the indicator of mean itch intensity can be used as the outcome of this meta-analysis, while other indicators were dropped because the size of included studies was less than three. The measurement of mean itch intensity was rated by a visual analogue scale (VAS) or a questionnaire about pruritus.

### 2.4. Data Extraction and Management

The authors of the reports were contacted to clarify any differences for obviously repeated studies. If the author could not be contacted, the first published study was deemed to be original. RCTs, which lack sufficient and consistent data, allow changes in net calculation of the outcomes and their variances from the baseline to the endpoint. Two reviewers (Yu C. and Lv Z. T.) selected the articles independently, and any discrepancies between reviewers were resolved through discussion with a third reviewer (Zhang P.) until a consensus was reached.

### 2.5. Data Synthesis and Analysis

The effectiveness of acupuncture therapy for itch was calculated as differences with continuous variable between placebo acupuncture, no treatment group, and acupuncture therapy by using Review Manager Software 5.2 (The Cochrane Collaboration). Heterogeneity was evaluated via the chi-square test and the tau^2^ test. A fix effects model was employed when the studies in the group were sufficiently alike (*P* > 0.10); otherwise, a random effects model was used. *Z* Score was calculated to test the overall effect, with significance set at *P* < 0.05.

## 3. Results

### 3.1. Literature Search Results

**Figure 1:**
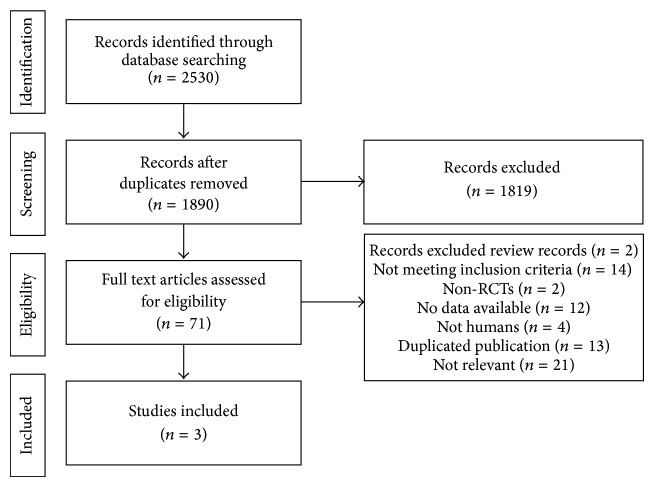


An initial search of RCTs yielded 2530 potential literature citations, including 2454 English studies and 76 Chinese studies, and 640 duplicated articles were deleted. After screening titles and abstracts, 71 potentially relevant studies were selected and retrieved for a full-text assessment. Of the remaining 71 studies, 2 studies were review records; 14 articles did not meet the inclusion criteria because their main therapy was not acupuncture; 2 studies were excluded because they were not RCTs; 12 studies had no data available; 4 studies' objects were not humans; 13 articles were duplicates; and 21 studies were not relevant. Finally, 3 studies [17, 19, 23] met our inclusion criteria and the process of study selection was shown in Figure 1.

### 3.2. The Characteristics of the Included Trials

All the included 3 studies were published as full text between 2005 and 2011 from Taiwan and Germany. Additionally, 2 studies of included samples were from the same laboratory and author. All the characteristics of the included studies were shown in Table 1.

### 3.3. Meta-Analysis Results

**Figure 2:**
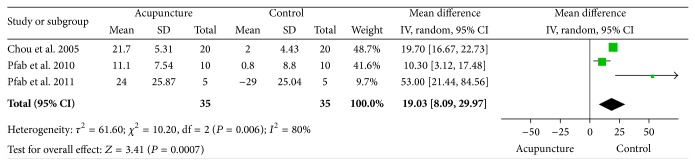


The analysis of 3 studies [17, 19, 23] was focused on the effect of acupuncture on itch intensity in 35 study subjects and 35 controls, including placebo acupuncture and no treatment. Heterogeneity test (Tau^2^ = 61.60, *χ* ^2^ = 10.20, df (degrees of freedom) = 2, *P* = 0.006, and *I* ^2^ = 80%) illustrated that the heterogeneity was unaccepted, so we performed this analysis by using random effect model as follows. The summary mean difference (MD) was 19.03 (95% CI: [8.09, 29.97], *Z* = 3.41, and *P*(*Z*) = 7 × 10^−4^) by using random effect model (Figure 2). Significant estimate was observed to support that acupuncture could be effective to inhibit itch intensity.

### 3.4. Publication Bias Analysis

**Figure 3:**
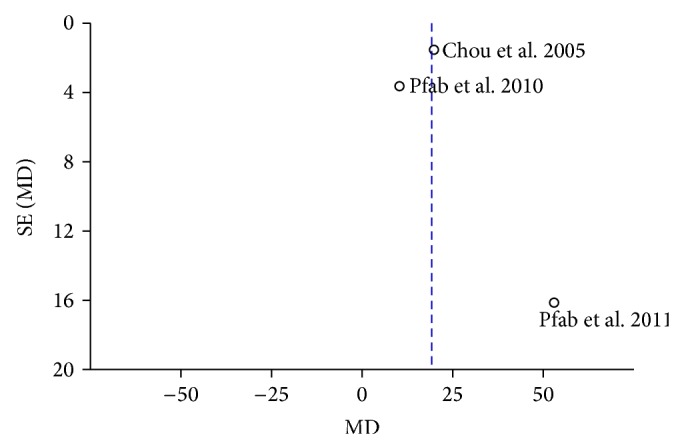


Funnel plot was used to check for the existence of publication bias. Because the sample size of this meta-analysis was quite small, the funnel plots indicated that the publication bias existed in the 3 included studies (Figure 3).

## 4. Discussion

### 4.1. Summary of Evidence

In this systematic review, we have shown the meta-analysis of therapeutic effect that applies acupuncture therapy to treat itch-related disease by analyzing all the collected data from three RCTs involving 70 individuals. The result revealed that acupuncture is effective to ameliorate itch intensity of itch-related disease; the *P* value (*P*(*Z*) = 7 × 10^−4^) is much smaller than the significant level (Figure 2).

### 4.2. Mechanism of Acupuncture Therapy

Itch can originate in the peripheral nervous system (dermal or neuropathic) or in the central nervous system (neuropathic, neurogenic, or psychogenic) [2, 24]. A large amount of people suffers from chronic itch such as those having psoriasis, eczema, and other chronic allergic skin diseases. Chronic itch also can indicate other health problems, such as liver and gall diseases, endocrine dyspraxia, metabolic diseases, chronic nephritis, and uremia [2]. It has been reported that acupuncture reduced itch and itch-evoked activation in the insula, putamen, premotor, and prefrontal cortical areas. Neither antihistamine nor placebo acupuncture could reduce itch or alter itch-related brain response [20]. Since greater itch reduction following acupuncture was associated with greater reduction in putamen response, which is a region responsible for motivation and habitual behavior underlying the urge to scratch, this region may be very critical in central mechanism of acupuncture's antipruritic effects [20].

Furthermore, it has been demonstrated that acupuncture and electroacupuncture (EA) stimulation are effective to treat pruritus if administered to affected dermatomes or adjacent dermatomes and this effect may be due to the antipruritic effect of kappa-opioid receptor activated by high-frequency EA stimulation [25]. Another study suggested that cold stimulation at LI11 attenuated drug-induced scratching behavior in mice and the mechanism may be mediated by transient receptor potential (TRP) channel-related pathway [26]. Even though many studies have reported certain mechanism about acupuncture therapy to ameliorate itch, further investigations to explain the mechanism of acupuncture therapy treating itch are still needed.

### 4.3. Limitations

There are still several limitations in this meta-analysis. Firstly, the number of studies and subjects included in this meta-analysis is still small. We found only one trial comparing acupuncture and pharmacological therapy, which is insufficient to evaluate the efficacy of acupuncture on itch. Secondly, all the included studies were from Taiwan and Germany, but itch is a worldwide symptom of a variety of diseases. Thirdly, although we used precise methods for study search, study inclusion, data extraction, and data analysis to minimize the bias, the heterogeneity between individual studies was also considered statistically significant. Last but not least, though we discovered numerous studies focusing on acupuncture treating itch-related disease in the process of screening studies, the rating scales of itch are different. For these reasons, we exclude numerous studies with high heterogeneity for using different rating scales in this meta-analysis.

### 4.4. Suggestion for Future Research

Firstly, more RCTs comparing the effect of acupuncture therapy with placebo acupuncture or pharmacological therapy on itch are needed. Secondly, it is also necessary to design three-armed RCTs, which can provide negative control and positive control of acupuncture simultaneously. Thirdly, the medical scientists all over the world should pay more attention to evaluate the efficacy of acupuncture on itch. What is more, the international rating scale of itch, as well as its evaluation standard, needs to be put forward and unified from international institutes of health as soon as possible. The implementation of this proposal would not only avoid the waste of resources but also provide more valued evidence for evidence-based medicine of acupuncture treating itch. Thus, in order to further effectively improve the treatment of itch, we suggest that all the medical researchers would adopt this suggestion without hesitation.

## 5. Conclusion

In conclusion, our systematic review suggests that acupuncture is effective for ameliorating itch intensity in itch-related diseases. However, this conclusion needs to be confirmed by more studies based on various ethnic samples in the future. Meanwhile, the unified evaluation scale of itch from international institutes of health should be put forward as soon as possible. Understanding neurobiological mechanism underlying antipruritic effects of acupuncture will significantly enhance the application of novel therapies to reduce itch.

## Figures and Tables

**Table 1:** The characteristics of the 3 included studies.

Author	Number of experiments	Number of control	Intervention method	Intervention location	Itch-related disease	Control method
Chou et al. [17]	20	20	Acupuncture	Quchi	Uremic pruritus	PA
Pfab et al. [19]	10	10	Acupuncture	Quchi, Xuehai	Atopic eczema	PA + NT
Pfab et al. [23]	5	5	Acupuncture	Quchi, Hegu, Zusanli, Xuehai	Atopic eczema	NT

Note: PA refers to placebo acupuncture and NT refers to no treatment.
